# Intestinal *Klebsiella pneumoniae* enhances neuroinflammation and seizure susceptibility in a pentylenetetrazole-induced acute seizure model

**DOI:** 10.3389/fnins.2026.1850015

**Published:** 2026-09-17

**Authors:** Junqiang Li, Yan Wang, Weiwen Cai, Sirui Chen, Lan Zhang, Tiancheng Wang

**Affiliations:** 1 Department of Neurology, The Second Hospital & Clinical Medical School, Lanzhou University, Lanzhou, Gansu, China; 2 Cuiying Biomedical Research Center, The Second Hospital & Clinical Medical School, Lanzhou University, Lanzhou, Gansu, China; 3 Department of General Surgery, The Second Hospital & Clinical Medical School, Lanzhou University, Lanzhou, Gansu, China

**Keywords:** epilepsy, *Klebsiella pneumoniae*, neuroinflammation, pentylenetetrazole, seizure susceptibility

## Abstract

Epilepsy is characterized by recurrent seizures and is driven in part by neuroinflammation-induced disruption of neuronal excitation-inhibition (E/I) balance. However, the upstream factors triggering microglial activation remain unclear. Here, we identify gut-enriched *Klebsiella pneumoniae* (*K. pneumoniae*) in patients with epilepsy and demonstrate that its derived metabolites activate microglial inflammatory responses while inducing neuronal oxidative stress and apoptosis. In a mouse model combining intestinal colonization with *K. pneumoniae* and pentylenetetrazole (PTZ)-induced seizures, we show that this bacterium exacerbates seizure susceptibility, enhances neuroinflammation, and disrupts hippocampal E/I balance. Mechanistically, *K. pneumoniae* may promote epileptogenesis through metabolite-driven, TNF-dependent inflammatory signaling, leading to aberrant microglial activation. Collectively, these findings establish a mechanistic link between gut microbiota-derived metabolites and epilepsy and identify *K. pneumoniae* as a potential therapeutic target.

## 1 Introduction

Epilepsy is a neurological disorder characterized by recurrent seizures, and its pathogenesis is closely associated with an imbalance between neuronal E/I. Among the contributing mechanisms, neuroinflammation has been recognized as a key upstream driver of this imbalance (Xu et al., 2025). Microglia, the principal immune effector cells of the central nervous system, play a central role in this process. Upon aberrant activation, microglia release pro-inflammatory cytokines, including TNF-α, IL-1β, and IL-6, which modulate synaptic transmission, enhance neuronal excitability, and thereby promote seizure generation (Salter and Stevens, 2017; Vezzani et al., 2011). Furthermore, accumulating evidence indicates that inflammatory mediators such as IL-1β can directly disrupt glutamate-GABA metabolism and receptor expression, leading to a breakdown of E/I balance (Geng et al., 2025). Despite the well-established role of neuroinflammation in epilepsy, the upstream triggers driving aberrant microglial activation remain largely undefined.

The gut-brain axis has recently emerged as a critical pathway linking the peripheral environment to central nervous system function. The gut microbiota can influence brain function through multiple mechanisms, including inflammatory signaling, metabolite production, and modulation of neurotransmitter pathways, and has been implicated in a range of neurological disorders (O’Riordan et al., 2025; Zhang et al., 2025). In epilepsy, accumulating evidence demonstrates that patients exhibit marked alterations in gut microbial composition, characterized by microbial dysbiosis, changes in both α and β-diversity, and an increased relative abundance of pro-inflammatory taxa (He and Zhang, 2024; McCafferty et al., 2022; Mousavi et al., 2025). These findings suggest that the gut microbiota may serve as an important source of peripheral inflammatory signals contributing to epilepsy-associated neuroinflammation.

Notably, clinical and experimental studies have shown that certain opportunistic pathogens are enriched in the gut microbiota of patients with epilepsy and may influence seizure susceptibility by modulating host immune and inflammatory responses (Lin et al., 2021). However, emerging evidence indicates that the effects of the gut microbiota on the host are not solely dependent on microbial colonization perse, but are largely mediated by microbiota-derived metabolites (Shin et al., 2015). As key mediators of host-microbe communication, these metabolites can enter the systemic circulation and directly or indirectly regulate immune responses and central nervous system function. Previous studies have demonstrated that microbial metabolites can modulate neuroinflammation through multiple pathways, including immune cell activation, cytokine production, and alterations in blood brain barrier permeability (Xu et al., 2024). In the context of epilepsy, such effects may exacerbate neuroinflammation and disrupt neuronal E/I balance, thereby promoting seizure activity. Nevertheless, the role of specific bacteria-derived metabolites in epileptogenesis and their underlying mechanisms remain poorly understood.

Here, we identify a significant enrichment of *K. pneumoniae* in the gut microbiota of patients with epilepsy. We further demonstrate that metabolites derived from *K. pneumoniae* activate microglial inflammatory responses and inhibit neuronal proliferation while inducing oxidative stress-associated apoptosis. *In vivo*, using a mouse model combining intestinal colonization with *K. pneumoniae* and PTZ-induced acute seizures, we show that this bacterium exacerbates seizure susceptibility, enhances neuroinflammatory responses, and disrupts hippocampal E/I balance. Mechanistically, our findings suggest that *K. pneumoniae* may promote epileptogenesis through metabolite-driven, TNF-dependent inflammatory signaling.

## 2 Materials and methods

### 2.1 Study cohort

This study was approved by the Ethics Committee of The Second Hospital & Clinical Medical School, Lanzhou University, and all the participants were informed. In total, 8 epilepsy patients (EP) and 8 healthy controls (HC) were included. The characteristics of the patients are summarized in Table 1. The inclusion criteria for patients with epilepsy were as follows: (1) Age > 18 years with a disease duration of at least 2 years. (2) Patients had not received anti-epileptic drug (AEDs) treatment before fecal sample collection. (3) No antibiotic use within the past 2 months. (4) No probiotic supplementation within the past week. (5) No consumption of spicy foods within the past week. (6) No diarrhea or severe constipation within the past week. (7) Female participants not pregnant or menstruating at the time of the study. The exclusion criteria were: (1) History of abdominal surgery within the past year. (2) Individuals with obesity (BMI ≥ 30). (3) Chronic smokers or regular alcohol consumers. (4) Major recent life changes. (5) History of gastrointestinal disorders.

**TABLE 1:** The characteristics of the patients and controls.

Variable	EP (*n* = 8)	HC (*n* = 8)	*P-*value
Age (years)	42.3 (28.9–51.3)	39.6 (33.0–59.3)	0.963
Gender			1.00
Male	4 (50.0%)	4 (50.0%)	
Female	4 (50.0%)	4 (50.0%)	
BMI (kg/m^2^)	21.38 ± 6.451	22.02 ± 5.573	0.790
Dietary habits			1.00
Grain-based diet	5 (62.5%)	5 (62.5%)	
Meat/dairy-based diet	3 (37.5%)	3 (37.5%)	
Epilepsy subtype classification			
Focal epilepsy	6 (75.0%)		
Generalized epilepsy	2 (25.0%)		
Seizure frequency			
<1 episode/month	4 (50.0%)		
≥1 episode/month	4 (50.0%)		
Disease duration, years			
2 to <4	5 (62.5%)		
4–7	3 (37.5%)		

### 2.2 Fecal sample collection and 16S rRNA gene sequencing

Fresh fecal samples were collected from patients with epilepsy and healthy controls, immediately transferred into sterile tubes, and stored at −80°C until further analysis. Total genomic DNA was extracted from fecal samples using a commercial DNA extraction kit according to the manufacturer’s protocol. The V3-V4 hypervariable regions of the bacterial 16S rRNA gene were amplified by PCR. Sequencing libraries were then constructed and subjected to paired-end sequencing on the Illumina NovaSeq platform. Raw sequencing data were processed through stringent quality control and chimera removal to obtain high-quality sequences for subsequent bioinformatic analyses.

### 2.3 Bacterial culture and preparation of culture supernatants

The *Klebsiella* (B32) and *Escherichia* (X48) strains used in this study were previously isolated from fecal samples of patients with epilepsy, identified, and preserved in our laboratory. The bacterial strains were inoculated into Brain Heart Infusion (BHI) medium and cultured at 37°C under aerobic conditions in a tri-gas incubator for 4 days. After incubation, the cultures were centrifuged at 7,500 *g* for 10 min at 4°C to collect the supernatants. The collected supernatants were then filtered through a 0.22 μm membrane to obtain sterile culture supernatants, which were used for subsequent cell treatment experiments.

### 2.4 Cell culture

BV2 microglial cells and HT22 hippocampal neuronal cells were cultured in DMEM supplemented with 10% fetal bovine serum (FBS) and 1% penicillin-streptomycin. HMC3 cells were cultured in MEM supplemented with 10% FBS and 1 × Antibiotics-Antimycotic. Cells were maintained in a humidified incubator at 37°C with 5% CO_2_. Cells in the logarithmic growth phase were used for all subsequent experiments.

### 2.5 RNA extraction and real-time quantitative PCR

Total RNA was extracted from cells or tissues using an RNA extraction kit (Vazyme, RC112) according to the manufacturer’s protocol. Equal amounts of RNA were reverse-transcribed into complementary DNA (cDNA) using a reverse transcription kit (Bio-Rad, 1708891). Real-time quantitative PCR (RT-qPCR) was performed using SYBR Green PCR Master Mix (Bio-Rad, 1725124) on a real-time PCR system. The relative mRNA expression levels of target genes were normalized to the internal reference gene Gapdh and calculated using the 2^∧^−ΔΔCt method. All experiments were performed in triplicate. The primer sequences for all genes are listed in Table 2.

**TABLE 2:** Primer sequences for the genes of interest are listed.

Name	Primer
Mouse Gapdh	FW: GCCTCCTCCAATTCAACCCTT
	RV: TGTCTACGGGACGAGGAAAC
Mouse Tnf-α	FW: CCCTCACACTCAGATCATCTTCT
	RV: GCTACGACGTGGGCTACAG
Mouse Il-1β	FW: GAAATGCCACCTTTTGACAGTG
	RV: TGGATGCTCTCATCAGGACAG
Mouse Il-6	FW: TCTATACCACTTCACAAGTCGGA
	RV: GAATTGCCATTGCACAACTCTTT
Mouse Gad1	FW: CACAGGTCACCCTCGATTTTT
	RV: ACCATCCAACGATCTCTCTCATC
Mouse Gad2	FW: TCCGGCTTTTGGTCCTTCG
	RV: ATGCCGCCCGTGAACTTTT
Mouse Ccl2	FW: TTAAAAACCTGGATCGGAACCAA
	RV: GCATTAGCTTCAGATTTACGGGT
Mouse Cxcl1	FW: CTGGGATTCACCTCAAGAACATC
	RV: CAGGGTCAAGGCAAGCCTC
Mouse Cxcl2	FW: CCAACCACCAGGCTACAGG
	RV: GCGTCACACTCAAGCTCTG
Mouse Cxcl10	FW: CCAAGTGCTGCCGTCATTTTC
	RV: GGCTCGCAGGGATGATTTCAA
Mouse Nfkb	FW: ATGGCAGACGATGATCCCTAC
	RV: TGTTGACAGTGGTATTTCTGGTG
Mouse Ocln	FW:TTGAAAGTCCACCTCCTTACAGA
	RV: CCGGATAAAAAGAGTACGCTGG
Total bacterial	340F: ACTCCTACGGGAGGCAGCAGT 514R: ATTACCGCGGCTGCTGGC
*K. pneumoniae*	K.p F: CTAAAACCGCCATGTCCGATTTAA K.p R: TTCCGAAAATGAGACACTTCAGA

### 2.6 Preparation of conditioned medium

BV2 microglial cells were seeded in 6-well plates at a density of 5 × 10^5^ cells per well and cultured in DMEM supplemented with 10% FBS to allow cell attachment. Cells were then treated with 10% *K. pneumoniae* supernatant or BHI medium for 24 h. Following treatment, the culture supernatants were collected, centrifuged to remove cell debris, and filtered through a 0.22 μm sterile membrane to obtain cell-free conditioned medium for subsequent experiments.

### 2.7 Cell viability assay using CCK-8

Cell proliferation was assessed using the Cell Counting Kit-8 (CCK-8) assay. HT22 cells were seeded in 96-well plates at a density of 2 × 10^3^ cells per well and cultured to allow cell attachment. Cells were then treated with 50% conditioned medium derived from BV2 cells pretreated with BHI medium and *K. pneumoniae*, and incubated for 24, 48, 72, 96 and 120 h, respectively. Subsequently, one-tenth the volume of CCK-8 reagent (Servicebio, G4103) was added to each well, followed by incubation at 37°C for 1 h. The absorbance at 450 nm was measured using a microplate reader. Each experimental condition was performed in triplicate.

### 2.8 EdU incorporation, oxidative stress and apoptosis assays

For EdU incorporation, oxidative stress and apoptosis assays, HT22 cells were treated with 50% conditioned medium derived from BV2 cells pretreated with BHI medium and *K. pneumoniae* for 24 h. Cell proliferation was assessed using an EdU assay kit (Beyotime, C0071S) according to the manufacturer’s protocol. Intracellular reactive oxygen species (ROS) levels were measured using a DCFH-DA fluorescent probe (Servicebio, G1706), with cells incubated at 37°C for 30 min in the dark, washed with serum-free medium, and fluorescence intensity detected using a fluorescence microplate reader or flow cytometry. Cell apoptosis was evaluated using an Annexin V-FITC/PI apoptosis detection kit (Multi sciences, AP105), followed by flow cytometric analysis after staining according to the manufacturer’s protocol.

### 2.9 *K. pneumoniae* colonization

Specific pathogen-free male C57BL/6 mice (8–10 weeks old) were randomly assigned to two groups. Mice experiments were performed under specific pathogen-free (SPF) conditions. Mice were housed in individually ventilated cages at 22 ± 2°C with 50%–60% relative humidity under a 12 h light/12 h dark cycle. Standard laboratory chow was provided *ad libitum*, and autoclaved water was provided *ad libitum* except during specific experimental interventions. To deplete the gut microbiota, mice received an antibiotic cocktail in the drinking water consisting of clindamycin (0.2 mg/mL), moxifloxacin (0.2 mg/mL), cefdinir (0.2 mg/mL) and linezolid (0.6 mg/mL) for 10 consecutive days. Following microbiota depletion, mice in the experimental group were orally gavaged with 200 μL of a *K. pneumoniae* suspension (1 × 10^9^ CFU/mL) every 2 days, whereas control mice received an equal volume of BHI medium. The gavage regimen was maintained for 14 days. At the end of the treatment period, fecal samples were collected for DNA extraction to verify successful intestinal colonization of *K. pneumoniae*. Total bacterial abundance in fecal samples was first quantified using universal 16S rRNA gene primers (UniF 340/UniR 514) for normalization. The relative abundance of *K. pneumoniae* was then determined using species-specific primers (*K.p* F/*K.p* R) (Barbier et al., 2020). This approach enabled accurate evaluation of *K. pneumoniae* colonization and ensured the establishment of a stable gut colonization model.

### 2.10 Pentylenetetrazol-induced acute seizure model and behavioral assessment

The acute seizure model was established by intraperitoneal injection of PTZ (Sigma, 54-95-5). PTZ was administered at a dose of 12.5 mg/kg every 10 min until mice exhibited generalized tonic-clonic seizures or a cumulative dose of approximately 100 mg/kg was reached (eight injections in total). Seizure latency and behavioral severity were recorded and analyzed according to the Racine scale. All behavioral assessments were performed in a blinded manner to minimize subjective bias (Su et al., 2025).

### 2.11 Statistical analysis

Statistical analyses were performed using SPSS (IBM, Armonk, NY, USA) and GraphPad Prism (GraphPad Software, San Diego, CA, USA). Comparisons among more than two groups were conducted using one-way analysis of variance (ANOVA), while non-parametric tests were applied for data that did not follow a normal distribution. Data are presented as mean ± standard error of the mean (SEM). Statistical significance was defined as *p < 0.05, ^**^p < 0.01, ^***^p < 0.001 and ^****^p < 0.0001.

## 3 Results

### 3.1 Altered gut microbial diversity and composition in epilepsy

To investigate differences in gut microbiota between HC and patients with EP, we collected fecal samples from 8 healthy individuals and 8 patients with epilepsy and performed 16S rRNA sequencing (Figure 1A). α-diversity analysis revealed a significant reduction in both the Chao1 and Shannon indices in the EP group compared with HC, indicating decreased microbial richness and diversity in patients with epilepsy (Figures 1B, C). Principal coordinates analysis (PCoA) demonstrated a clear separation between the EP and HC groups, suggesting distinct microbial community structures, which was further confirmed by PERMANOVA analysis (*P* < 0.05) (Figure 1D).

**FIGURE 1:**
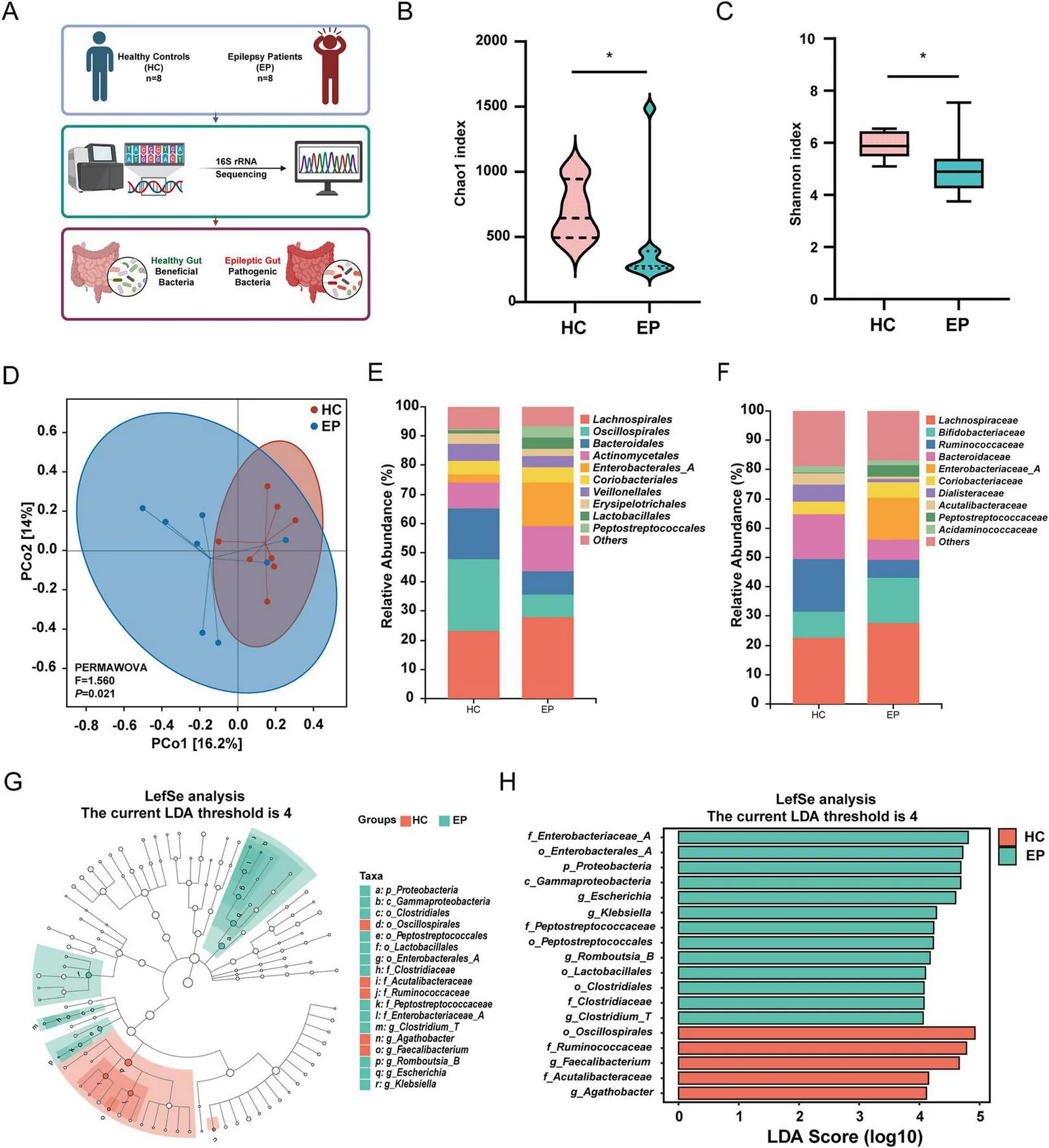
Gut microbiota dysbiosis in patients with epilepsy. **(A)** Study design for fecal sample collection and 16S rRNA gene sequencing in healthy controls (HC, *n* = 8) and patients with epilepsy (EP, *n* = 8). **(B,C)** Alpha diversity analysis showing the Chao1 index **(B)** and Shannon index **(C)** in the EP groups. **(D)** Principal coordinates analysis (PCoA) based on microbial community composition showing a clear separation between the HC and EP groups; PERMANOVA analysis indicated a significant difference between groups (*P* < 0.05). **(E,F)** Relative abundance of differential taxa between the HC and EP groups at the order **(E)** and family **(F)** levels. **(G)** LEfSe cladogram showing differentially enriched bacterial taxa between the HC and EP groups. **(H)** LDA score distribution (LDA > 4) highlighting enrichment of Escherichia and Klebsiella in the EP group. Data are presented as mean ± SEM (*p < 0.05).

At the taxonomic level, the microbial composition differed markedly between groups. Specifically, at the order level, Lachnospirales and Enterobacterales_A were increased, whereas Oscillospirales and Bacteroidales were decreased in the EP group. At the family level, Lachnospiraceae and Enterobacteriaceae_A were enriched, while Bacteroidaceae and Ruminococcaceae were reduced (Figures 1E, F). LEfSe analysis (LDA > 4) further identified *Escherichia* and *Klebsiella* as significantly enriched at the genus level in the EP group (Figures 1G, H).

Collectively, these results indicate a pronounced remodeling of the gut microbiota in patients with epilepsy, characterized by enrichment of potentially pathogenic taxa (e.g., Enterobacteriaceae and *Klebsiella*) alongside depletion of beneficial commensals, indicating an overall shift toward a pro-inflammatory microbial profile.

### 3.2 *K. pneumoniae* markedly induces inflammatory responses in BV2 microglial cells in a dose-dependent manner.

Recent evidence suggests that the pathogenesis of epilepsy is not only associated with aberrant neuronal discharges but is also closely linked to persistent neuroinflammatory responses. Meanwhile, gut microbiota and their metabolites, as important peripheral modulators, are increasingly recognized to play critical roles in regulating central nervous system inflammation (Park et al., 2025; Villasana-Salazar and Vezzani, 2023). To identify potential bacterial strains associated with epilepsy, we first assessed the pro-inflammatory effects of *Escherichia* and *Klebsiella* strains that were significantly enriched in the feces of patients with epilepsy on microglial cells (Figure 2A). Based on this screening, two candidate strains previously isolated and preserved in our laboratory, X48 (*Escherichia*) and B32 (*Klebsiella*), were selected for further analysis. Phylogenetic analysis of their sequencing data showed that strain B32 clustered with *Klebsiella pneumoniae* strain DSM 30104 within a well-supported *Klebsiella* clade, whereas strain X48 was assigned to the *Escherichia* lineage.

**FIGURE 2:**
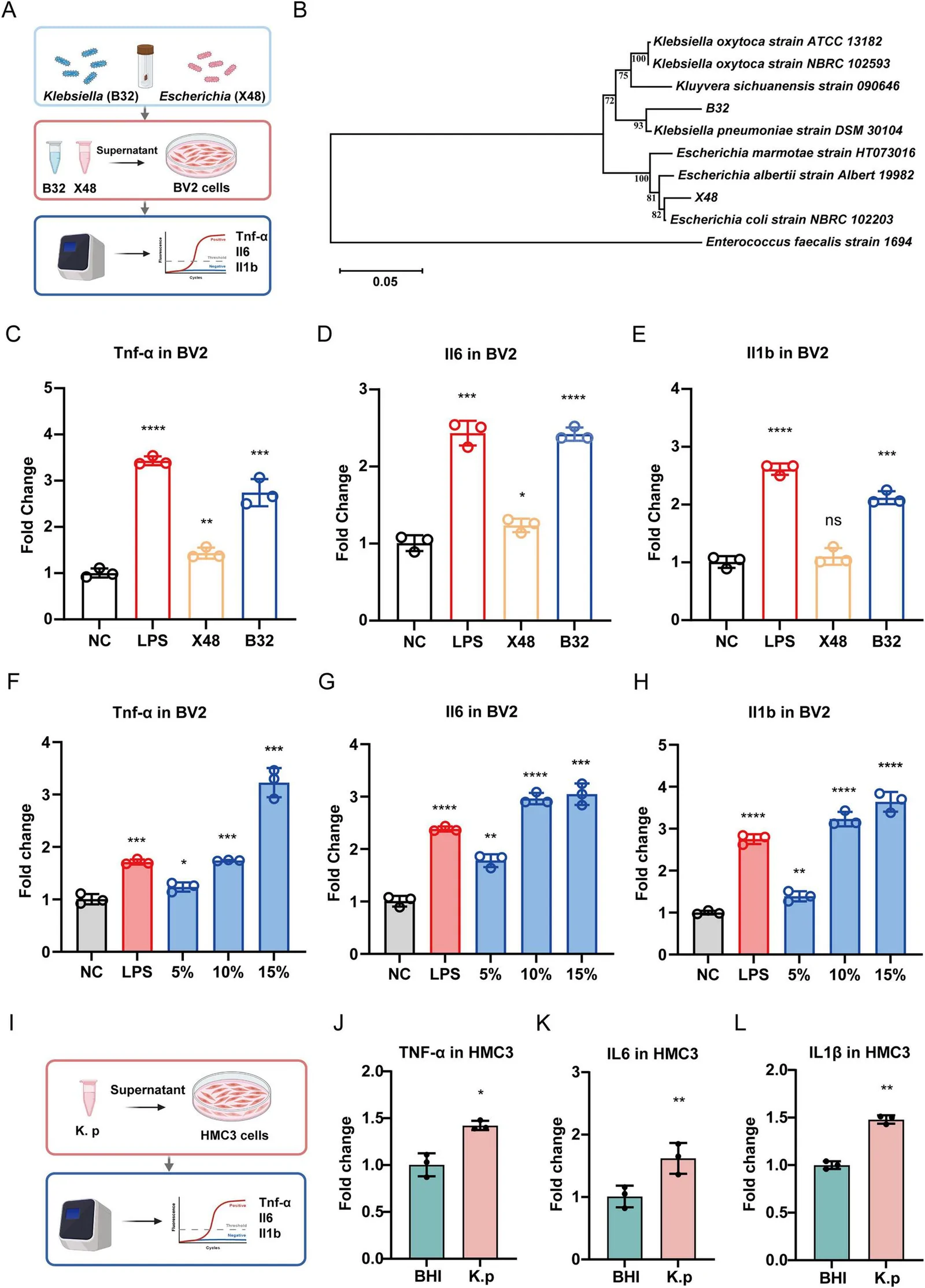
*Klebsiella pneumoniae* exhibits enhanced pro-inflammatory activity in BV2 microglial cells. **(A)** Experimental workflow for screening *Escherichia* and *Klebsiella* strains isolated from fecal samples of patients with epilepsy for their pro-inflammatory effects on BV2 microglial cells. **(B)** Phylogenetic analysis identifying B32 as *Klebsiella pneumoniae* and X48 as *Escherichia coli.* **(C–E)** mRNA expression of Tnf-α **(C)**, Il-6 **(D)**, and Il-1β **(E)** in BV2 cells treated with supernatants from *Klebsiella pneumoniae* (B32) and *Escherichia coli* (X48). **(F–H)** mRNA expression of Tnf-α **(F)**, Il-6 **(G)**, and Il-1β **(H)** in BV2 cells treated with increasing concentrations of *K. pneumoniae* supernatant. **(I)** Experimental workflow for transcript-level analysis of HMC3 cells treated with *K. pneumoniae*. **(J–L)** mRNA expression of TNF-α **(J)**, IL-6 **(K)**, and IL-1β **(L)** in HMC3 cells treated with increasing concentrations of *K. pneumoniae* supernatant. Data are presented as mean ± SEM (*p < 0.05, **p < 0.01, ***p < 0.001, ****p < 0.0001).

We next assessed the effects of culture supernatants derived from *K. pneumoniae* (B32) and *Escherichia coli* (X48) on inflammatory responses in BV2 microglial cells. Both supernatants significantly increased the mRNA expression of Tnf-α and Il-6, while the *K. pneumoniae* supernatant additionally induced a marked upregulation of Il-1β expression (Figures 2C–E), indicating a stronger pro-inflammatory capacity of *K. pneumoniae*. On this basis, BV2 cells were further treated with increasing concentrations of *K. pneumoniae* supernatant. The transcriptional levels of Tnf-α, Il-6 and Il-1β increased in a concentration-dependent manner (Figures 2F–H), demonstrating a clear dose–response relationship. To further determine whether this inflammatory response could also be observed in human microglial cells, we performed additional validation using HMC3 cells (Figure 2I). Consistent with the findings in BV2 cells, *K. pneumoniae* treatment significantly increased the mRNA levels of TNF-α, IL-6, and IL-1β in HMC3 cells (Figures 2J–L).

Taken together, these findings indicate that the supernatant derived from *K. pneumoniae* robustly induces inflammatory activation in BV2 microglial cells in a dose-dependent manner. Consistent with these results, *K. pneumoniae* treatment also increased the mRNA expression of TNF-α, IL-6, and IL-1β in HMC3 human microglial cells. These findings suggest that soluble factors derived from *K. pneumoniae* can promote inflammatory transcriptional responses in both murine and human microglial cell models, thereby potentially contributing to microglia-mediated neuroinflammation.

### 3.3 *K. pneumoniae* suppresses neuronal proliferation and induces oxidative stress and apoptosis via microglial activation

Microglia, as the primary immune-regulatory cells of the central nervous system, play a pivotal role in mediating neuronal injury and dysfunction through their inflammatory responses (Di Pietro et al., 2026; Yang et al., 2025). To determine whether *K. pneumoniae*-induced microglial activation affects neuronal behavior, we examined the effects of BV2-derived conditioned medium on HT22 neurons. Treatment with BV2 supernatant significantly suppressed HT22 cell proliferation (Figure 3A). Consistently, EdU incorporation assays revealed a marked reduction in the proportion of DNA-synthesizing cells in the treated group (Figures 3B–C), indicating that *K. pneumoniae*-conditioned microglia indirectly inhibit neuronal proliferation.

**FIGURE 3:**
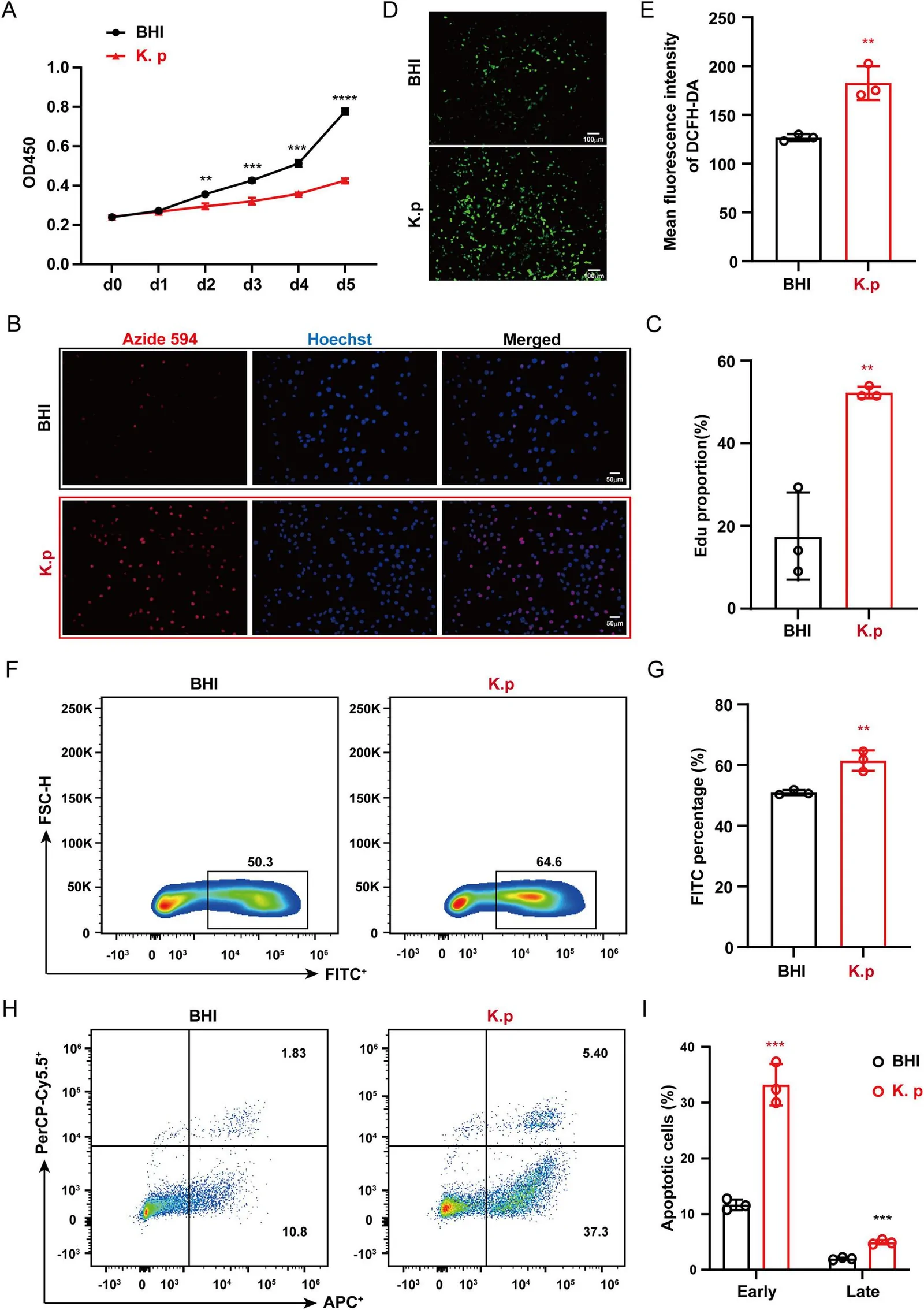
Conditioned medium from *K. pneumoniae*-stimulated BV2 cells induces neuronal dysfunction. **(A)** Reduced viability of HT22 neurons following treatment with conditioned medium. **(B,C)** Representative EdU staining **(B)** and quantification **(C)** showing decreased proliferation. **(D,E)** Increased intracellular ROS levels shown by fluorescence imaging **(D)** and quantification **(E)**. **(F,G)** Flow cytometric analysis **(F)** and quantification **(G)** of oxidative stress-positive cells. **(H,I)** Flow cytometric analysis **(H)** and quantification **(I)** of apoptotic cells. Data are presented as mean ± SEM. Statistical significance was determined as indicated in the corresponding panels (*p < 0.05, **p < 0.01, ***p < 0.001, ****p < 0.0001).

Oxidative stress is a key mechanism underlying neuronal damage and dysfunction (Houldsworth, 2024; Liu et al., 2025). We therefore assessed oxidative stress levels in HT22 cells following treatment. Fluorescence microscopy showed a pronounced increase in intracellular reactive oxygen species (ROS) in the treated group compared with controls (Figure 3D), which was further confirmed by quantitative fluorescence analysis (Figure 3E). In addition, flow cytometry analysis demonstrated a significant increase in the proportion of oxidatively stressed cells in the treated group (Figures 3F–G), indicating that *K. pneumoniae*-conditioned microglia markedly enhance oxidative stress in neurons.

Apoptosis represents a major pathological feature of neuronal injury and degeneration (Cai et al., 2025). To further evaluate the impact of *K. pneumoniae*-conditioned microglia on neuronal apoptosis, we performed flow cytometric analysis. The results showed that both early and late apoptotic cell populations were significantly increased in the treated group compared with controls (Figures 3H–I), indicating that *K. pneumoniae*-conditioned microglia promote neuronal apoptosis.

Collectively, these findings demonstrate that *K. pneumoniae*-derived factors, through microglial activation, not only suppress neuronal proliferation but also exacerbate oxidative stress and promote apoptosis, further indicating a microglia-mediated inflammatory mechanism plays an important role in neuronal injury.

### 3.4 *K. pneumoniae* promotes neuroinflammation and worsens seizure phenotypes *in vivo*

To investigate the potential pathogenic role of *K. pneumoniae* in epilepsy, we established a mouse model integrating antibiotic pretreatment, *K. pneumoniae* colonization, and PTZ-induced seizures (Figure 4A), and evaluated its effects on seizure susceptibility and inflammatory responses *in vivo*. *K. pneumoniae* gavage did not significantly affect body weight throughout the experimental period (Figure 4B), indicating that bacterial colonization had no overt impact on general physiological status. Fecal analysis confirmed successful and stable intestinal colonization of *K. pneumoniae* following gavage (Figure 4C). Following colonization, PTZ was administered to induce seizures, and behavioral assessments were performed. Compared with control mice, *K. pneumoniae*-treated mice exhibited significantly increased seizure severity, as reflected by higher seizure scores (Figure 4D), along with a markedly reduced seizure latency (Figure 4E). These findings indicate that *K. pneumoniae* colonization enhances seizure susceptibility and exacerbates epileptic phenotypes *in vivo*.

**FIGURE 4:**
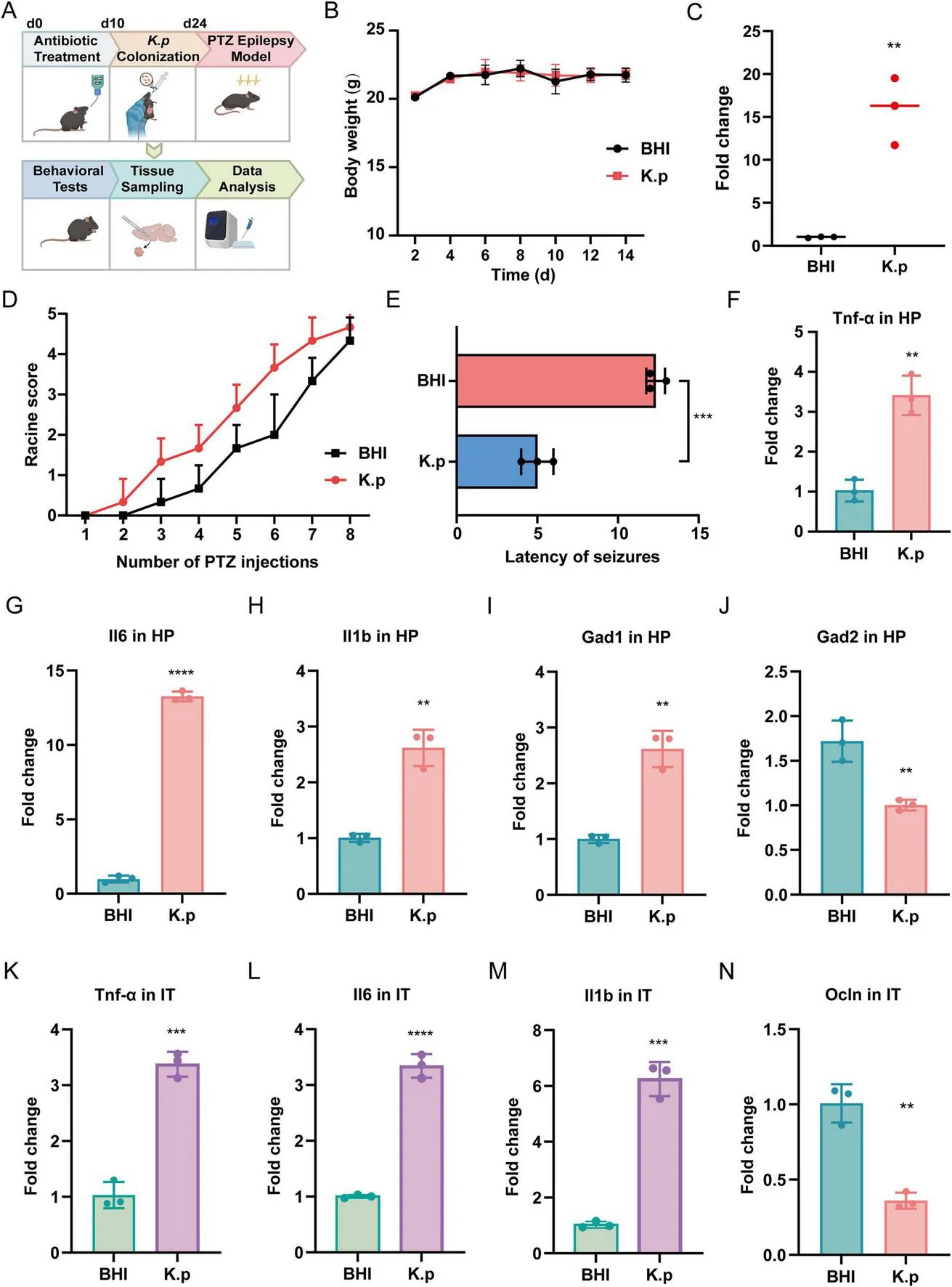
*Klebsiella pneumoniae* colonization exacerbates seizure susceptibility and promotes systemic inflammation *in vivo*. **(A)** Experimental design for antibiotic pretreatment, *K. pneumoniae* colonization, and PTZ-induced seizures. **(B)** Body weight remains unchanged during the experimental period. **(C)** qPCR confirms successful intestinal colonization of *K. pneumoniae*. **(D)** Increased seizure severity following PTZ injection. **(E)** Reduced latency to the first seizure in *K. pneumoniae*-treated mice. **(F–J)** Hippocampal expression of Tnf-α **(F)**, Il-6 **(G)**, Il-1β **(H)**, Gad1 **(I)** and Gad2 **(J)**. **(K–N)** Intestinal expression of Tnf-α **(K)**, Il-6 **(L)**, Il-1β **(M)** and Ocln **(N)**. Data are presented as mean ± SEM (**p < 0.01, ***p < 0.001, ****p < 0.0001).

We next examined gene expression profiles associated with neuroinflammation and inhibitory neurotransmission in the hippocampus. *K. pneumoniae*-treated mice displayed significantly elevated mRNA levels of pro-inflammatory cytokines, including Tnf-α, Il-6 and Il-1β (Figures 4F–H), suggesting that *K. pneumoniae* colonization is associated with enhanced hippocampal inflammatory responses in the context of PTZ-induced seizures. Concurrently, Gad1 expression was upregulated, whereas Gad2 expression was downregulated (Figures 4I–J), suggesting a disruption of GABAergic neurotransmission and an imbalance in excitatory-inhibitory signaling. In addition, inflammatory and barrier-related gene expression was assessed in intestinal tissues. The transcriptional levels of Tnf-α, Il-6 and Il-1β were significantly increased in the gut of *K. pneumoniae*-treated mice (Figures 4K–M), indicating the presence of peripheral inflammation. Notably, the mRNA expression of the tight junction marker Ocln was reduced in the *K. pneumoniae* group compared with the control group, as determined by RT-qPCR (Figure 4N), suggesting that *K. pneumoniae* colonization may affect intestinal barrier-related gene expression.

Overall, these findings suggest that *K. pneumoniae* colonization is associated with enhanced hippocampal and peripheral inflammatory responses in the context of PTZ-induced seizures, accompanied by altered intestinal barrier-related gene expression and exacerbated seizure-related phenotypes.

### 3.5 *K. pneumoniae* promotes inflammatory activation through TNF signaling

Microglia, as the principal innate immune cells of the central nervous system, play a critical role in amplifying neuroinflammation and driving neuronal dysfunction through inflammatory signaling and cytokine release (Muller and Di Benedetto, 2025). To investigate the mechanisms by which *K. pneumoniae* contributes to epileptogenesis, we performed transcriptomic profiling of BV2 microglial cells following *K. pneumoniae* treatment (Figure 5A). Principal coordinates analysis (PCoA) revealed a clear separation between *K. pneumoniae*-treated and control groups (Figure 5B). Differential expression analysis identified 81 significantly altered genes, including 50 upregulated and 31 downregulated transcripts (Figure 5C), which was further summarized in the differential gene distribution analysis (Figure 5D).

**FIGURE 5:**
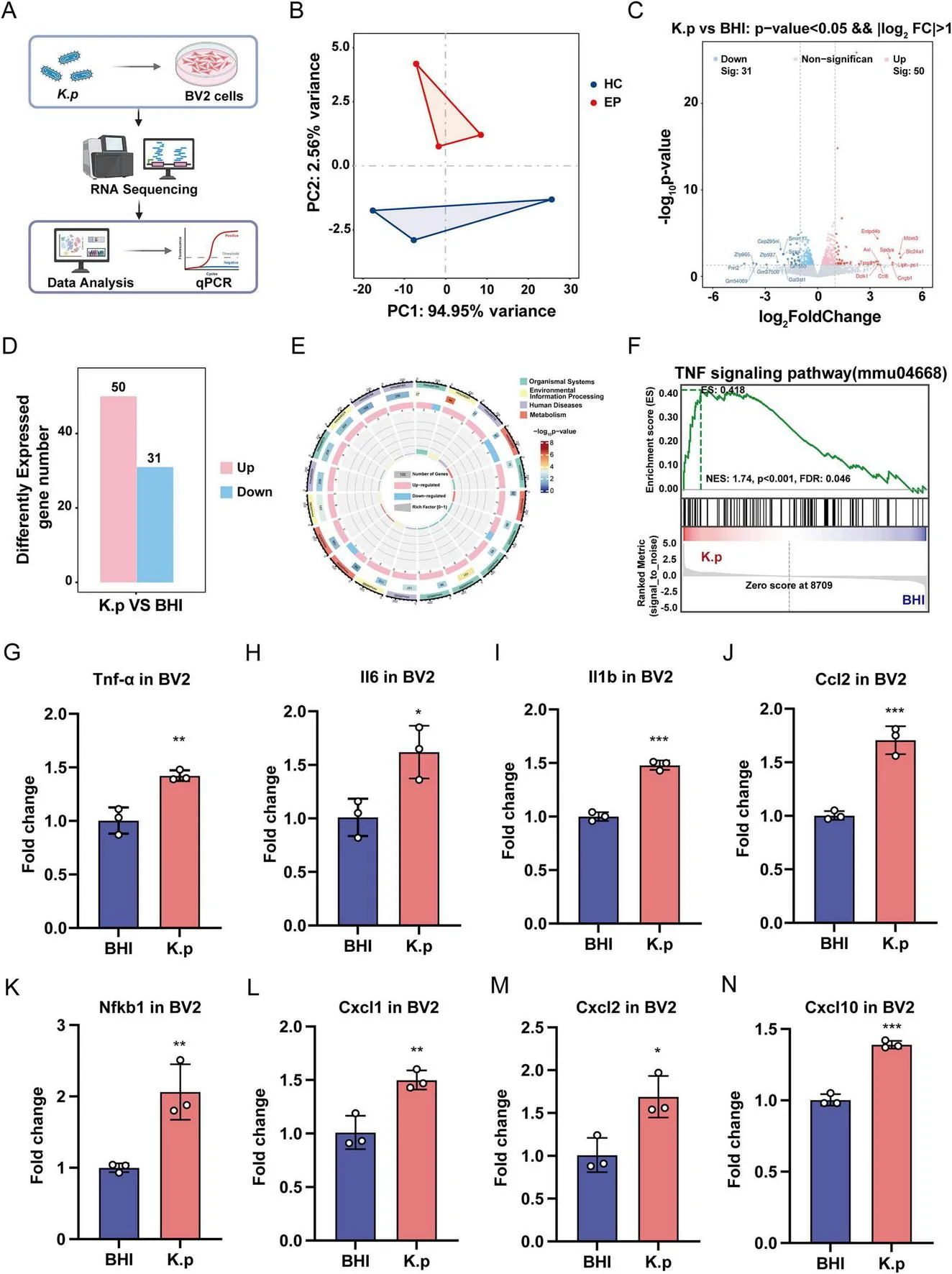
*Klebsiella pneumoniae* induces TNF-dependent inflammatory transcriptional reprogramming in BV2 microglia. **(A)** Experimental workflow for BV2 treatment and transcriptomic analysis. **(B)** PCoA reveals distinct clustering between *K. pneumoniae*-treated and control groups. **(C)** Volcano plot of differentially expressed genes. **(D)** Distribution of significantly upregulated and downregulated genes. **(E)** Circular visualization of transcriptomic features and functional annotation of differentially expressed genes. **(F)** GSEA demonstrates enrichment of the TNF signaling pathway. **(G–N)** Upregulation of Tnf-α**(G)**, Il-6 **(H)**, Il-1β **(I)**, Ccl2 **(J)**, Nfkb1 **(K)**, Cxcl1 **(L)**, Cxcl2 **(M)** and Cxcl10 **(N)** in BV2 cells following *K. pneumoniae* treatment. Data are presented as mean ± SEM (*p < 0.05, **p < 0.01, ***p < 0.001).

To further explore the functional relevance of these transcriptional changes, we performed Gene Ontology (GO) and Kyoto Encyclopedia of Genes and Genomes (KEGG) enrichment analyses. These analyses demonstrated that the differentially expressed genes were predominantly associated with inflammatory and immune-related processes (Figure 5E). Notably, the TNF signaling pathway was among the most significantly enriched pathways. Consistently, gene set enrichment analysis (GSEA) further confirmed robust activation of the TNF signaling pathway (Figure 5F), highlighting its central role in *K. pneumoniae*-induced transcriptional reprogramming.

To validate these findings, we examined the expression of key inflammatory mediators in BV2 cells. qRT-PCR analysis revealed that *K. pneumoniae* treatment significantly upregulated the mRNA levels of pro-inflammatory cytokines, including Tnf-α, Il-6 and Il-1β, as well as multiple chemokines and signaling-related genes, such as Ccl2, Nfkb1, Cxcl1, Cxcl2 and Cxcl10 (Figures 5G–N). These results collectively indicate that *K. pneumoniae* activates a broad inflammatory transcriptional programme in microglia, primarily via the TNF signaling axis, thereby contributing to the amplification of neuroinflammatory responses.

In summary, these results indicate that *K. pneumoniae* amplifies microglial inflammatory responses by activating the TNF signaling pathway and inducing a broad spectrum of cytokines and chemokines, thereby contributing to the exacerbation of neuroinflammation.

## Discussion

Increasing evidence indicates that patients with epilepsy exhibit marked gut microbiota dysbiosis compared with healthy individuals, characterized by reduced microbial diversity and profound alterations in community structure (Peng et al., 2018). Emerging studies suggest that such dysbiosis may influence seizure susceptibility through immune-inflammatory signaling and modulation of neurotransmitter systems (Kwack et al., 2024; Li et al., 2025; Yang et al., 2024). In the present study, by integrating clinical cohort analysis, *in vitro* cellular experiments and *in vivo* animal models, we systematically investigated the role of *K. pneumoniae* in epilepsy and its underlying mechanisms. We demonstrate that the gut microbiota of patients with epilepsy is significantly altered, with enrichment of Enterobacteriaceae and *Klebsiella*. Functionally, *K. pneumoniae*-derived supernatant robustly induced pro-inflammatory cytokine expression in BV2 microglia in a dose-dependent manner, while conditioned medium from activated microglia impaired HT22 neuronal proliferation, increased oxidative stress and promoted apoptosis. *In vivo*, intestinal colonization with *K. pneumoniae* shortened seizure latency and increased seizure severity, accompanied by enhanced inflammatory responses in both hippocampal and intestinal tissues, as well as dysregulation of neurotransmitter-related genes. Transcriptomic analysis further indicated that *K. pneumoniae* predominantly drives microglial activation through the TNF signaling pathway. Collectively, these findings suggest that *K. pneumoniae* promotes neuroinflammation via the gut-brain axis and increases seizure susceptibility.

Neuroinflammation is widely recognized as a central mechanism in epileptogenesis. Microglia, as the principal immune effector cells in the central nervous system, play a critical role in regulating neuroinflammatory responses and maintaining neuronal network homeostasis (Juca et al., 2024; Shi and Yong, 2025). Upon activation, microglia release pro-inflammatory cytokines such as TNF-α, IL-1β and IL-6, which enhance neuronal excitability and contribute to seizure initiation and progression (Ouyang et al., 2025; Zhao et al., 2025). In line with this, our data demonstrate that *K. pneumoniae* markedly upregulates these cytokines in BV2 cells and activates the TNF signaling axis, suggesting that soluble bacterial factors may act as upstream triggers of aberrant microglial activation. These findings provide direct evidence linking gut-derived microbial alterations to central neuroinflammation in epilepsy.

Beyond inflammatory activation, our study further highlights a pivotal intermediary role of microglia in mediating neuronal injury. Conditioned medium from *K. pneumoniae*-stimulated microglia significantly inhibited HT22 neuronal proliferation and induced intracellular ROS accumulation and apoptosis, indicating that microglia-derived inflammatory mediators can directly impair neuronal function. Previous studies have shown that the gut microbiota can regulate neurotransmitter metabolism, particularly affecting GABA and glutamate pathways, thereby influencing the excitation/inhibition (E/I) balance (Glinert et al., 2022; Park and Kim, 2021). Consistently, we observed that *K. pneumoniae* colonization resulted in upregulation of Gad1 and downregulation of Gad2 in the hippocampus. Given that GAD65 (encoded by Gad2) is essential for synaptic GABA release, its downregulation may weaken inhibitory neurotransmission, disrupt E/I balance and ultimately increase network excitability. These results suggest that *K. pneumoniae* may promote epileptogenesis through a dual mechanism involving inflammatory amplification and neurotransmitter imbalance.

The gut-brain axis represents a bidirectional communication network linking gut microbiota with central nervous system function. Microbial components and metabolites can modulate microglial activation via immune signaling and systemic circulation, thereby shaping neuroinflammatory states (Aburto and Cryan, 2024; Agirman et al., 2021; Arneth, 2025; Ohara and Hsiao, 2025). In this study, *K. pneumoniae* not only induced central inflammation but also markedly enhanced intestinal inflammation, indicating a coordinated peripheral-central inflammatory response. This finding underscores the critical contribution of gut inflammation to epilepsy pathophysiology and highlights the regulatory role of the gut microbiota in neurological disorders. From a translational perspective, while conventional anti-epileptic therapies primarily target neuronal hyperexcitability, microbiota-targeted interventions-such as probiotics, fecal microbiota transplantation and dietary modulation-are increasingly being explored. Targeting *K. pneumoniae* or its associated inflammatory pathways may therefore represent a promising therapeutic strategy for epilepsy.

Several limitations should be acknowledged. First, the relatively small clinical sample size warrants validation in larger cohorts. Therefore, the association between intestinal *K. pneumoniae* enrichment and epilepsy should be interpreted as preliminary, and future studies with larger cohorts and more comprehensive clinical metadata are needed. Second, the specific metabolites or virulence factors mediating the effects of *K. pneumoniae* remain to be identified. Because *K. pneumoniae* is a Gram-negative bacterium, the potential contribution of LPS or other bacterial components in the sterile culture supernatant cannot be excluded and should be further investigated in future studies. Third, this study employed an acute PTZ-induced seizure model; future investigations in chronic epilepsy models are required to confirm these findings. Integration of multi-omics approaches, including metagenomics, metabolomics and single-cell sequencing, will be essential to further dissect the mechanisms underlying *K. pneumoniae*-mediated gut-brain axis regulation.

In conclusion, our study provides a comprehensive framework linking clinical microbial alterations to molecular mechanisms, demonstrating that *K. pneumoniae* promotes seizure susceptibility by activating microglial inflammatory responses and disrupting neurotransmitter homeostasis. These findings offer new insights into the microbiological basis of epilepsy and identify potential targets for therapeutic intervention.

## Data Availability

The raw 16S rRNA gene sequencing data have been deposited in GSA under accession number CRA047577 and the BV2 transcriptomic sequencing data have been deposited in NCBI SRA under BioProject accession number PRJNA1505693.
